# Podoconiosis in East and West Gojam Zones, Northern Ethiopia

**DOI:** 10.1371/journal.pntd.0001744

**Published:** 2012-07-17

**Authors:** Yordanos B. Molla, Sara Tomczyk, Tsige Amberbir, Abreham Tamiru, Gail Davey

**Affiliations:** 1 Brighton and Sussex Medical School, Falmer, Brighton, United Kingdom; 2 International Orthodox Christian Charities, Addis Ababa, Ethiopia; 3 International Orthodox Christian Charities, Debre Markos, Ethiopia; Ghana Health Service, Ghana

## Abstract

**Background:**

Podoconiosis is a neglected tropical disease (NTD) that is prevalent in red clay soil-covered highlands of tropical Africa, Central and South America, and northern India. It is estimated that up to one million cases exist in Ethiopia. This study aimed to estimate the prevalence of podoconiosis in East and West Gojam Zones of Amhara Region in northern Ethiopia.

**Methodology/Principal Findings:**

A cross-sectional household survey was conducted in Debre Eliyas and Dembecha *woredas* (districts) in East and West Gojam Zones, respectively. The survey covered all 17,553 households in 20 *kebeles* (administrative subunits) randomly selected from the two *woredas*. A detailed structured interview was conducted on 1,704 cases of podoconiosis identified in the survey.

**Results:**

The prevalence of podoconiosis in the population aged 15 years and above was found to be 3.3% (95% CI, 3.2% to 3.6%). 87% of cases were in the economically active age group (15–64 years). On average, patients sought treatment five years after the start of the leg swelling. Most subjects had second (42.7%) or third (36.1%) clinical stage disease, 97.9% had mossy lesions, and 53% had open wounds. On average, patients had five episodes of acute adenolymphangitis (ALA) per year and spent a total of 90 days per year with ALA. The median age of first use of shoes and socks were 22 and 23 years, respectively. More men than women owned more than one pair of shoes (61.1% vs. 50.5%; χ^2^ = 11.6 p = 0.001). At the time of interview, 23.6% of the respondents were barefoot, of whom about two-thirds were women.

**Conclusions:**

This study showed high prevalence of podoconiosis and associated morbidities such as ALA, mossy lesions and open wounds in northern Ethiopia. Predominance of cases at early clinical stage of podoconiosis indicates the potential for reversing the swelling and calls for disease prevention interventions.

## Author Summary

Podoconiosis is non-infectious elephantiasis that affects barefoot people that have prolonged exposure to red clay soil. It is common in tropical Africa, central America and northern India. Podoconiosis presents as bilateral below knee swelling. Podoconiosis can be both prevented and controlled by consistently washing feet, wearing shoes, and using antiseptics and emollients. This survey is the biggest conducted to date in Ethiopia: 17,553 households in East and West Gojam Zones of northern Ethiopia were included, and 1,704 patients were identified. We interviewed patients in detail about manifestations of acute attacks (painful inflammation of the foot and leg with swollen lymph nodes and fever), clinical disease stage, treatment seeking, foot washing and shoe wearing practices. We found the prevalence of podoconiosis to be 3.3%. Most patients were farmers, uneducated and within the economically active age group. There was no gender difference in occurrence of podoconiosis and in foot washing practices. The onset of leg swelling and the age of first shoe wearing were similar. We also found delayed treatment seeking and many days confined to bed during acute inflammatory episodes. We conclude that podoconiosis imposes a huge burden in northern Ethiopia.

## Introduction

Podoconiosis is a non- infectious elephantiasis distinct from lymphatic filariasis that affects barefoot individuals exposed to red clay soil of volcanic origin. In particular, podoconiosis is prevalent among barefoot subsistence farmers that live and work in these areas [1]. Even though the pathogenesis of the diseases has not yet been investigated in depth, it is believed to be caused by fine particles in the soil that penetrate the skin and induce an inflammatory reaction in the lymphatic system [2]. The disease results in bilateral progressive swelling of the lower legs, usually limited below the level of the knees. Based on the disease progression, podoconiosis is classified into five stages where the first and second stages have swelling limited below ankle which is either reversible over night (stage one) or not (stage two). The third stage of the disease has water bag like or nodular swelling above the level of the ankle. The fourth stage entails above knee swelling whereas the fifth stage involves joint fixation as a result of surrounding soft tissue overgrowth [3]. Podoconiosis can be identified clinically in endemic areas (above 1200 m) without the need to do laboratory tests because it usually presents with bilateral and asymmetric swelling on the lower limbs with rare groin involvement unlike the lymphatic form of elephantiasis [4]. Similarly, the nervous system is intact in podoconiosis and there is neither loss of sensation nor thickened nerves or trophic ulcers unlike leprosy [5], [6]. Podoconiosis can be prevented, early forms of the disease can be treated, disease progression can be curbed and the disease can potentially be eliminated as a public health burden with low technology but effective measures such as washing feet with soap and water on a regular basis and wearing protective shoes consistently [7], [8], [9].

High prevalence of podoconiosis has been reported in many parts of highland Africa: Ethiopia [10], Cameroon [11], Rwanda [12], Burundi, Sudan [13], Uganda [14], Tanzania [15], Kenya [16], the islands of Bioko, Sao Tome and Principe [17], and Equatorial Guinea [18]. Up to one million cases are estimated to exist in Ethiopia, of whom one-third belong in the economically productive age group [10]. Recent studies in southern and western Ethiopia estimated the prevalence of podoconiosis to be 5.5% [10] and 2.8% [19], respectively.

Although the Amhara Region in northern Ethiopia is one of the regions burdened by podoconiosis in specific highland districts, there have been no recent studies to assess the prevalence, clinical features, and socio-economic burden of the disease. To our knowledge, the only two studies that provided data on the prevalence of the disease in Amhara region date back more than 40 years [20], [21]. In June 2010, International Orthodox Christian Charities (IOCC), a non-government organization, started the first podoconiosis program in East Gojam Zone, Amhara Region. The program aims to address podoconiosis prevention, awareness, and care and support activities. To strengthen the services provided in East Gojam Zone and scale up to West Gojam Zone, there was an evident need for baseline data to direct the public health interventions. Therefore, the aim of this study was to assess the burden of podoconiosis in East and West Gojam Zones of Amhara Regional State, northern Ethiopia. Specifically, the study aimed to answer questions including, but not limited to, overall, gender- and age-specific prevalence; manifestations of acute attack episodes (acute adenolymphangitis or ALA: painful inflammation of the foot and leg with swollen lymph nodes and fever); clinical disease stage; treatment seeking behavior; and foot washing and shoe wearing practices of patients.

## Methods

### Ethics statement

Ethical clearance was obtained from the Amhara Regional Health Bureau. Support letters were obtained from East and West Gojam Zonal Health Departments and Woreda Health Offices. Oral informed consent was obtained from each study participant after reading the written consent form to them, since most of the respondents were not able to read and write. The interviewers confirmed the participant's oral consent by signing on the respective consent form for each interview as per the guideline of the ethical review Board of the Amhara Regional Health Bureau. When children aged under 18 years (the legal age for giving consent for research in Ethiopia) were encountered, consent was obtained from their parents or guardians. The use of verbal consent was approved by the ethical review committee because the majority of the study participants cannot read and write.

### Study design and study area

This study was a cross-sectional community-based house-to-house survey. The study was conducted in East and West Gojam Zones of Amhara Regional State. All villages (*Ketena*) in known podoconiosis-endemic *kebeles* (the lowest level government administrative structure in Ethiopia) were included in the house to house case enumeration. Identification of the study area was based on a report by IOCC's podoconiosis treatment center, written in 2010 summarizing information from key local informants. The study participants were residents of the selected *kebeles* and podoconiosis cases in all households with podoconiosis.

### Sampling procedure and sample size determination

The Ethiopian administrative structure is organized hierarchically, with multiple Zones in each Region. Each Zone contains multiple Woredas (equivalent to districts). Each Woreda contains Kebeles and there are villages with multiple households in each Kebele. A convenience non-random sampling method was used to select two Zones. A list of *woredas* (government administrative units equivalent to districts) in East and West Gojam Zones, known for the presence of podoconiosis based on expert opinion and key informants, was prepared. Second, a random sampling technique was applied to select two *woredas*, one from each Zone. Third, *kebeles* from each of these two *woredas* were sampled randomly proportional to their population size in each *woreda*. A total of 20 *kebeles* from the two *woredas* in East Gojam and West Gojam Zones (7 *kebeles* from East Gojam and 13 *kebeles* from West Gojam) were selected. All households in the selected *kebeles* were assessed for the presence of podoconiosis cases through interviews with the household head followed by clinical examination of cases by community health extension workers (HEW). Households with podoconiosis cases were included for individual podoconiosis case interviews by clinical nurses. In households where there was more than one podoconiosis patient, all patients were interviewed, and physical examination and measurement of leg circumference were done.

### Data collection process

Data collection was done house-to-house by trained HEWs supervised by nurses that work in the respective *woredas*. The HEWs were responsible for house-to-house enumeration of podoconiosis cases and the nurses were responsible for supervising the activities of the HEWs and the detailed assessment of podoconiosis cases (i.e., interviewing and physical examination of patients). All HEWs and nurses received training from the team of researchers before performing data collection. The training consisted of techniques and approaches for obtaining informed consent from prospective participants, interviewing techniques, podoconiosis diagnostic features, clinical staging according to a standard method [3], assessment of ALA, measurement of leg circumference (the largest circumference between the levels of the ankle and knee measured using a tape, to a precision level of the nearest centimeter [3]), assessment of presence of open wounds, and features that differentiate lymphoedema and leg swelling resulting from podoconiosis from other diseases such as leprosy and filarial elephantiasis. Furthermore, these data collectors were trained on how to advise patients to wear shoes and wash their feet to control disease progression at the end of every interview.

A pre-test of the actual data collection process and the data collection tools was conducted immediately after the training of the data collectors. The pretest was done in two *kebeles* (one in West Gojam *Woreda* and the other in East Gojam *Woreda*) which were not included in the main survey. The pretests were evaluated in terms of (i) organization of the fieldwork and coordination between the team of investigators, supervisor nurses and HEWs; (ii) ability of the HEWs to effectively conduct the census and complete the questionnaires; (iii) ability of the supervisor nurses to correctly diagnose and stage podoconiosis, and identify ALA symptoms; (iv) completeness, skip patterns, flow and clarity of the questionnaire. At the end of the pre-test, the trainees brought back the data they collected to the training centre. The completed questionnaires were checked by the trainers. Discussion on the challenges the trainees faced during data collection and on the data collection tools was held and the questionnaires were revised accordingly.

### Data collection tools

The data collection tool was a structured questionnaire. The questionnaire was developed in English, translated into Amharic and back translated into English to check consistency. The questionnaire was sub-sectioned thematically into socio-demographic characteristics, podoconiosis history, clinical features, treatment-seeking behavior, sources of water, walking practices, foot hygiene and shoe wearing practices.

### Data processing and analysis

Data were entered and analysed using the Statistical Package for Social Sciences (SPSS) software v.17.0. The overall prevalence of podoconiosis was calculated as the ratio of the number of patients with podoconiosis to the total population surveyed aged 15 years and over. Statistical significance was tested using the Chi-squared test or t-test as appropriate. The level of significance was set at α of 0.05.

## Results

### Socio-demographic characteristics

A total of 17,553 households with 51,017 members aged 15 years and above were included in the present survey. Of the surveyed households, 9.7% had one or more podoconiosis patients. A total of 1,319 podoconiosis patients that provided consent and were available during the interview participated in the detailed patient interview. Characteristics of these patients are presented in Table 1. Almost all patients were in the age group 15–64 (the age group that includes economically active individuals in Ethiopia), did not read and write, and were farmers. More women than men patients were divorced (22.5% vs. 3.6%, χ^2^ = 102.3, p<0.0001).

**Table 1:** Characteristics of interviewed patients (n = 1,319), Debre Eliyas and Dembecha *Woredas*, East and West Gojam Zones, northern Ethiopia.

Characteristics	Category	Men (n = 670)	Women (n = 649)	Total (n = 1,319)
Patients' average age in years (n = 1,314)	Mean (SD)	45.6 (14.6)	42.9 (13.8)	44.3 (14.3)
Age distribution in years (n = 1,314), n (%)	<15 years	3 (0.4)	6 (0.9)	9 (0.7)
	15–24	39 (5.8)	47 (7.2)	86 (6.5)
	25–34	110 (16.4)	128 (19.7)	238 (18)
	35–44	161 (24.0)	152 (23.4)	313 (23.7)
	45–54	151 (22.5)	160 (24.7)	311 (23.6)
	55–64	123 (18.4)	102 (15.7)	225 (17.1)
	>64 years	79 (11.8)	53 (8.2)	132 (10.0)
	15–64 years	584 (87.2)	589 (90.8)	1173 (88.9)
Patients' marital status, n (%)	Single	53 (7.9)	44 (6.8)	97 (7.4)
	Married	567 (84.6)	348 (53.6)	915 (69.4)
	Divorced	24 (3.6)	144 (22.2)	168 (12.7)
	Separated	1 (0.1)	5 (0.8)	6 (0.5)
	Widowed	14 (2.1)	100 (15.4)	114 (8.6)
Years lived in the area (n = 1,283)	Mean (SD)	42.3 (16.5)	37.1 (16.5)	39.7 (16.7)
Patients' level of education. (n = 1,307), n (%)	Cannot read and write	488 (72.8)	558 (86.0)	1046 (79.3)
	Primary level	80 (11.9)	51 (7.9)	131 (9.9)
	Secondary level	8 (1.2)	11 (1.7)	19 (1.4)
	College/university	0	1 (0.2)	1 (0.0008)
	Informal education	41 (6.1)	5 (0.8)	46 (3.5)
	Unknown level	47 (7)	17 (2.6)	64 (4.9)
Patients' occupation (1,303), n (%)	Farming	601 (89.7)	382 (58.9)	983 (74.5)
	Housewife	NA	129 (19.9)	129 (9.8)
	Daily laborer	24 (3.6)	40 (6.2)	64 (4.9)
	Student	19 (2.8)	17 (2.6)	36 (2.7)
	Waiter	0	10 (1.5)	10 (0.8)
	Selling local beverage	1 (0.1)	23 (3.5)	24 (1.8)
	Land rental	1 (0.1)	11 (1.7)	12 (0.9)
	Unemployed	2 (0.3)	5 (0.8)	7 (0.5)
	Handcraft and weaving	5 (0.7)	7 (1.1)	12 (0.9)
	Other*	8 (1.2)	17 (2.6)	25 (1.9)

*: Others: priest, begging, prostitute, shepherd, private business, retired and guard.

### Prevalence and clinical characteristics

The prevalence of podoconiosis was found to be 3.3% (95% CI = 3.2% to 3.6%) in people aged 15 years and above. The prevalence was 3.3% (95% CI = 3.1% to 3.6%) in Debre Eliyas and 3.4% (95% CI = 3.2% to 3.6%) in Dembecha *Woredas*. The male to female ratio was 0.98∶1. More than 87% of the patients fell within the 15 to 64 year age group, and less than 2% were under the age of 15 years (Table 2).

**Table 2:** Prevalence of podoconiosis (n = 88,879).

Category	Population surveyed in the age group ≥15 years#	Number of households surveyed	Number of cases identified	Prevalence in those aged ≥15 years	Men to women ratio
**Debre Eliyas.**	20,089	7,109	661	661/20,089 = 3.3%	362/299 (1.2∶1)
**Dembecha.**	30,531	10,436	1,042	1,042/30,531 = 3.4%	484/558(0.9∶1)
**Total**	51,017	17,553	1,704	1,704/51,017 = 3.3%	846/858(0.98∶1)

# **The proportion the population of 15 years and above, by sex, was calculated based on the Ethiopian Central Statistics Authority Report: 55.5% for Debre Eliyas** ***Woreda*** **, 58% for Dembecha** ***Woreda*** **, and 57.4% for Amhara Region** [**30**] **.**

The median age of onset of leg swelling was 22 years (range: 10 to 77 years). On average, patients sought treatment 5 years (SD = 6.9) after the start of the leg swelling, predominantly at health centers (39.8%) and from traditional healers (39.1%).

**Figure 1:**
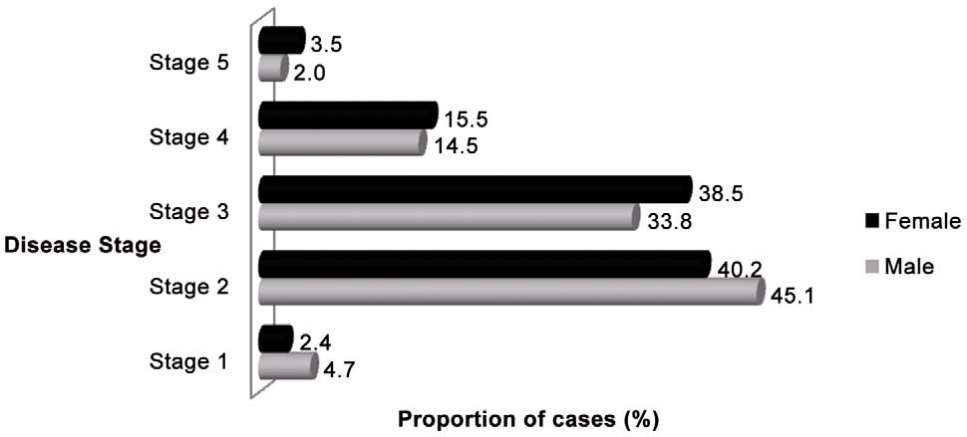
Clinical stages of podoconiosis among female and male patients in Debre Eliyas and Dembecha *woredas*, northern Ethiopia.

More study subjects had symmetric bilateral swelling than unilateral or asymmetric bilateral swelling. Most subjects had second or third clinical stage disease (Table 3 and Figure 1).

**Table 3:** Clinical stages of podoconiosis among the study subjects.

Clinical stage		Male (n = 611)	Female (n = 572)	Overall (n = 1,183)
Unilateral swelling, n (%)	Stage I	71 (11.6)	37 (6.5)	108 (9.1)
	Stage II	593 (97.1)	503 (87.9)	1,096 (92.6)
Bilateral and symmetric swelling, n (%)	Both legs stage I	26 (4.3)	12 (2.1)	38 (3.2)
	Both legs stage II	255 (41.7)	216 (37.8)	471 (39.8)
	Both legs stage III	147 (24.1)	172 (30.1)	319 (26.9)
	Both legs stage IV	68 (11.1)	63 (11.0)	131 (11.1)
Bilateral and asymmetric swelling, n (%)	One leg stage I, other leg stage II	18 (2.9)	12 (2.1)	30 (2.5)
	One leg stage I, other leg stage III	1 (0.2)	1 (0.2)	2 (0.2)
	One leg stage II, other leg stage III	59 (9.7)	47 (8.2)	106 (8.9)

Nearly all (97.9%) patients had mossy lesions and 53% had open wounds on at least one of their legs.

### Features of acute adenolymphangitis (ALA)

On average, patients had five episodes of ALA per year and had 90 ALA morbidity days per year. Among patients who had ALA episodes more frequently than once per month (n = 323), 73.1% had ALA during the two weeks prior to the date of interview. Similarly, 94.4% of the ALA episodes reported in the year prior to the interview happened in the most recent six months. Nearly half of the participants (49.8%) had ALA during the interview. Physical examination of the interviewees that had ALA showed that hot (49.8%) and tender (60.2%) swelling, and inguinal lymphadenopathy (62.1%) were common features (Table 4).

**Table 4:** Features of acute lymphadenitis (ALA) in podoconiosis patients.

Feature	Category	Male	Female	Overall
Leg circumference (n = 922), n (%)	<21	18 (2.0)	9 (1.0)	27 (2.9)
	21–25	147 (15.9)	123 (13.3)	270 (29.3)
	26–36	334 (36.2)	361 (39.2)	695 (75.4)
	37–47	50 (5.4)	59 (6.4)	109 (11.8)
	>47	2 (0.2)	1 (0.1)	3 (0.3)
Last time the patient had ALA, n (%)	Last 2 weeks (n = 323)	104 (32.2)	132 (40.9)	236 (73.1)
	Last 1 month (n = 557)	81 (14.5)	79 (14.2)	160 (28.7)
	Last 6 months (n = 557)	269 (48.3)	257 (46.1)	526 (94.4)
	Beyond 1 year (n = 392)	165 (42.1)	150 (38.3)	315 (80.4)
Sought treatment (n = 1,299), n (%)	Yes	325 (49.2)	311 (48.7)	636 (49.0)
	No	336 (50.8)	327 (51.3)	663 (51.0)
Season when symptoms of ALA get worse (n = 1,281), n (%)	Rainy and wet season	166 (25.7)	158 (24.9)	324 (25.3)
	Hot and dry season	358 (55.4)	340 (53.5)	698 (54.5)
	No specific season	122 (18.9)	134 (21.1)	256 (20.0)
ALA Precipitating factors, n (%)	Hard (laborious) work (n = 1,161)	167 (28.6)	168 (29.1)	335 (28.9)
	A little more than usual work (n = 992)	104 (21.1)	124 (24.8)	228 (23.0)
	Long walk (n = 992)	348 (70.6)	368 (73.7)	716 (72.2)
	*Mitch* (n = 167)	45 (51.7)	42 (48.3)	87 (52.1)
	Dust (n = 167)	10 (45.5)	12 (54.5)	22 (13.2)
Coping mechanism for ALA, n (%)	Resort to less exertion (n = 966)	234 (49.6)	193 (39.1)	427 (44.2)
	Stay in bed (n = 966)	237 (50.2)	300 (60.7)	537 (55.6)
	Using herb (*Hareg resa*) (n = 322)	36 (19.8)	30 (21.4)	66 (20.5)
	Using antibiotics (n = 322)	53 (29.1)	30 (21.4)	83 (25.8)
	Sleeping/nothing (n = 322)	27 (14.8)	25 (17.9)	52 (16.1)
	Washing (n = 322)	14 (7.7)	11 (7.9)	25 (7.8)
	Other (soaking in water, drinking alcohol, etc) (n = 322)	15 (8.2)	16 (11.4)	(9.6)

Half of the study subjects (49%) that had ALA in the past one year (n = 1,299) sought treatment for the pain. The commonest treatment facilities visited were health centers (28.7%) and traditional healers (29.4%). Among patients that went to other places for the treatment of ALA (n = 196), most (80.6%) went to *Tsebel* (holy water) places. Patients stated that on average they spent five days in bed during episodes of ALA.

Over half of the study participants (54.5%) said ALA commonly occurred during the hot and dry seasons, whereas 20% said the episodes were not season specific. The most common ALA precipitating factors mentioned by patients were long walks (72.2%), ‘*mitch*’ (effect of the sun inducing inflammation, 52.1%), laborious work (28.9%), and dust (13.2%). The most common coping measures employed by patients to reduce morbidity during episodes of ALA were staying in bed (55.6%), resorting to less laborious work (44.2%), use of antibiotics (25.8%) and *Hareg Resa* (a local herb that is boiled generating steam that is inhaled by patients believed to have *mitch*, 20.5%).

### Shoe-wearing, foot-washing and walking experience

The median ages of first use of shoes and socks in the area were 22 and 23 years, respectively. About 40% of the patients said that they had one pair of shoes. More men than women owned more than one pair of shoes (61.1% vs. 50.5%; χ^2^ = 11.6 p = 0.001). Most study participants suggested that they need three pairs of shoes per year.

The types of shoes worn by the study participants at the time of interview were covered hard plastic (33.3%) followed by canvas (20.9%) and *berebaso/gilet* (open sandals locally made from tires) (13.2%). During the interview, 23.6% of the respondents were observed to be barefoot of whom most (65.3%) were women. Men were more likely to wear open, non-protective shoes. There was no statistically significant association between gender and use of protective shoes (p = 0.92).

Based on patients' estimates, the average one-way walking time to the nearest water source for washing feet was 19 minutes (SD = 15.7). The average one-way walking time to the nearest field and nearest market were 20 minutes (SD = 14.9) and 54.5 minutes (SD = 50.1), respectively. On average, the respondents travelled to their nearest field and to market nine (SD = 10.9) and four (SD = 3.9) times per month, respectively.

On observation, 46.8% of patients had clean feet, 25.1% had dirty feet, 13.9% had cracked feet and 13.9% had both dirty and cracked feet. The reported average frequency of foot washing was seven times a week. There was no statistically significant difference between men and women in the frequency of foot washing (p = 0.12) and foot washing with soap (p = 0.33) (Table 5).

**Table 5:** Shoe wearing and foot washing experience of patients.

Characteristics	Category	Male	Female	Total
Average age at first shoes	Mean (SD)	25.4 (12.6)	24.9 (13.6)	25.2 (13.1)
Average age at first socks	Mean (SD)	25.6 (12.7)	22.4 (15.2)	24.3 (13.9)
Number of pairs of shoes owned (n = 1,168), n (%)	None	44 (7.3)	87 (15.4)	131 (11.2)
	One pair	218 (36.1)	236 (41.8)	454 (38.9)
	Two pairs	237 (39.2)	169 (26)	406 (34.8)
	Three pairs	83 (13.7)	58 (10.3)	141 (12.1)
	Four Pairs	18 (3.0)	13 (2.3)	31 (2.7)
	More than four pairs	4 (0.7)	1 (0.2)	5 (0.5)
Number of pairs of shoes needed (n = 1,177), n (%)	One pair	25 (4.1)	26 (4.6)	51 (4.3)
	Two pairs	171 (28.0)	171 (30.2)	342 (29.1)
	Three pairs	210 (34.4)	179 (31.6)	389 (33.1)
	Four pairs	151 (24.7)	142 (25.1)	293 (24.9)
Times when patients do not wear shoes (n = 1,170), n (%)	During farming	145 (24.1)	50 (8.8)	195 (16.7)
	During non-farming work	132 (22.0)	52 (9.2)	184 (15.7)
	At home	153 (25.5)	200 (35.2)	353 (30.2)
	I am usually barefoot	100 (16.6)	160 (28.2)	260 (22.2)
	I am always barefoot	71 (11.8)	106 (18.7)	177 (15.1)
Feet cleanliness at the time of interview (n = 1,287), n (%)	Clean and intact	331 (50.2)	274 (43.4)	605 (46.8)
	Dirty	147 (22.3)	177 (28)	324 (25.1)
	Cracked	80 (12.1)	99 (15.7)	179 (13.9)
	Dirty and cracked	99 (15.0)	80 (12.7)	179 (13.9)
Average frequency of foot washing per week	Mean (SD)	6.8 (2.1)	6.9 (2.2)	6.8 (2.1)
Use soap for washing feet (n = 1,302), n (%)	Yes	407 (61.9)	424 (65.9)	831 (63.8)
	No	250 (38.1)	219 (34.1)	469 (36.0)
Average frequency of foot washing with soap per week	Mean (SD)	5.2 (2.9)	5.1 (3.1)	5.2 (3.0)

## Discussion

This study presents the largest of all household surveys of podoconiosis in Ethiopia conducted over the past ten years. It is the first community-based study of podoconiosis in the Amhara region, the second largest region in Ethiopia. Men and women were equally affected by podoconiosis, as found in the Wolaita study (1∶0.98) [10] but in contrast to the Gulliso (1∶2.6) [19] and Ocholo (1∶4.2) [22] studies. The prevalence of podoconiosis in the present study area (3.3%) was higher than the recent reports for Gulliso *woreda* in western Ethiopia (2.8%) [19] but lower than that reported among long-term residents in Ocholo village in southern Ethiopia (5.1%) [22], among resettlement scheme residents in Illubabor, western Ethiopia (9.1%), among residents in Gera and Didessa towns in southern Ethiopia (8–10%) [23], among residents in Midakegn district, central Ethiopia [24], and in Wolaita, southern Ethiopia (5.5%) [10]. The prevalence falls within the earliest national estimate of 0.4% to 3.7% across fifty-six markets [20]. The prevalence of podoconiosis found in the present study was greater than the previous estimates in Gojam of 2.4% based on market counts by Oomen [20] and 2.3% based on school enquiry by Price [21] nearly four decades ago in Debre Markos, the capital of East Gojam Zone. These studies had limitations since they were based on market counts of cases and school enquiry of students from villages and were therefore likely to underestimate the overall prevalence. Nevertheless, the absence of decline in prevalence of podoconiosis after four decades clearly reflects that podoconiosis has remained neglected in the Amhara region.

Based on our findings, we reviewed the potential opportunities and challenges for primary and secondary prevention of podoconiosis in Gojam. Primary prevention consists of avoiding prolonged skin to soil contact by wearing protective shoes and socks. Secondary prevention requires regular foot hygiene, and the use of antiseptic soaks and emollients. Early evidence of effectiveness of this treatment in reducing leg circumference and disease stage and in improving quality of life has recently emerged from a small, uncontrolled follow-up study by Sikorski et. al [9]. Since most cases of podoconiosis in the present study were in the early stages of 2 and 3, secondary prevention is potentially possible in this area.

The study also revealed several challenges to primary and secondary prevention. First, there were serious deficiencies in protection against the soil with footwear. Suboptimal shoe-wearing behavior was reported even among those owning shoes, and observation at the time of interviews confirmed a substantial proportion of barefoot respondents. The types of shoes worn by most patients were not considered protective. Although men owned more pairs of shoes than women, there were similar low levels of shoe wearing and foot washing practice among men and women resulting in almost equal prevalence of disease. However, more advanced stages were seen among women. The similar average age of onset of leg swelling and age of first shoe wearing indicate prolonged contact with the soil and delay in protection.

Secondly, there was substantial continuous contact with irritant soil. In addition to daily work as subsistence farmers, the frequency of travel and walking distances to and from water sources, markets and fields considerably increased the duration of contact with the soil. Limited access to water (average round trip of 40 minutes) may explain poor foot hygiene as patients prioritize water for drinking and cooking before washing their feet. The average time spent travelling to get water in Gojam was greater than in Gulliso, western Ethiopia (average round trip of 20 minutes) and also greater than the WHO recommended maximum (30 minutes round trip) [25]. This will constitute a considerable challenge for primary and secondary prevention of podoconiosis in Gojam.

Thirdly, the long interval between onset of swelling and seeking treatment indicate an important delay in accessing secondary prevention to control disease progression. Most of the podoconiosis cases in our study were found in the highland areas away from the towns where most health centers are based. There was no podoconiosis treatment in Amhara Region before the establishment of the IOCC podoconiosis prevention and treatment center in June 2010. The patients who visited health centers for treatment may not have received appropriate treatment, forcing them to try other forms of support including traditional medicine. Previous studies have demonstrated health professionals' misconceptions surrounding podoconiosis and stigmatizing attitudes towards patients, so patients who attended formal health facilities may not have received treatment [26].

Fourthly, limited knowledge and misconceptions about the disease by the patients themselves may also act as a barrier against primary and secondary prevention by delaying treatment seeking behavior [27]. In Wolaita, adopting avoidant coping strategies led to patient isolation and reduced access to health care [28]. Similarly, linking ALA to a range of experiences including *mitch* resulted in poor treatment seeking and resorting to traditional medicine for a significant proportion of the patients.

This study reinforces recent evidence from Gulliso indicating the enormous burden imposed by ALA among podoconiosis patients [19]. The frequency of attacks, duration of pain and number of bed days due to ALA were all considerable. Physical examination of the interviewees reporting ALA at the time of interview confirmed the widespread frequency of the problem. Although our study did not compare the financial status of patients with unaffected people in the area, previous studies have shown the large economic burden imposed by podoconiosis [25]. This burden arises because of the concentration of the disease in the productive age group as seen in this study and the effect of ALA hampering productivity [10], [29].

In conclusion, the prevalence of podoconiosis in Gojam is almost identical to that described in the 1970s by Price and Ooman [20], [21] indicating that the disease has been almost entirely neglected since that time. Significant challenges remain in increasing protective shoe-wearing practices, improving access to water for washing, and encouraging earlier treatment seeking. However, given that early stages of disease were predominantly observed, if treatment is offered, it is likely to be effective.

## References

[1] Davey G (2008). Podoconiosis: let Ethiopia lead the way Ethiop.. J Health Dev.

[2] Davey G (2010). Podoconiosis, non-filarial elephantiasis, and lymphology.. Lymphology.

[3] Tekola F, Ayele Z, Haile Mariam D, Fuler C, Davey G (2008). Development and testing of a de novo clinical staging system for podoconiosis (endemic non-filarial elephantiasis).. Tropical Medicine and International Health.

[4] Desta K, Meskele A, Davey G (2007). Predictive value of clinical assessment of patients with podoconisis in an endemic community setting.. Trans R Soc Trop Med Hyg.

[5] Davey G, Tekola F, Newport MJ (2007). Podoconiosis: non-infectious geochemical elephantiasis.. Trans R Soc Trop Med Hyg.

[6] Desta K, Ashine M, Davey G (2007). Predictive value of clinical assessment of patients with podoconiosis in an endemic community setting.. Trans R Soc Trop Med Hyg.

[7] Davey G, Burridge E (2009). Community-Based Control of a Neglected Tropical Disease: The Mossy Foot Treatment and Prevention Association.. PLoS Negl Trop Dis.

[8] Price EW (1990). Podoconiosis: Non-filarial Elephantiasis. 1st ed.

[9] Sikorski C, Ashine M, Zeleke Z, Davey G (2010). Effectiveness of a Simple Lymphoedema Treatment Regimen in Podoconiosis Management in Southern Ethiopia: One Year Follow-Up.. PLoS Negl Trop Dis.

[10] Destas K, Ashine M, Davey G (2003). Prevalence of podoconiosis (endemic non-filarial elephantiasis) in Wolaitta, Southern Ethiopia.. Trop Doct.

[11] Wanji S, Tendongfor N, Esum M, Che JN, Mand S (2008). Elephantiasis of non-filarial origin (podoconiosis) in the highlands of north-western Cameroon.. Ann Trop Med Parasitol.

[12] Ruberanziza E, Mupfasoni D, Karibushi B, Rujeni N, Kabanda G (2009). Mapping of Lymphatic Filariasis in Rwanda.. J Lymphoedema.

[13] Price EW, Bailey D (1984). Environmental factors in the etiology of endemic elephantiasis of the lower legs in tropical Arica.. Trop Geogr Med.

[14] Onapa AW, Simonsen PE, Pederson EM (2001). Non-filarial elephantiasis in the Mt. Elgon area (Kapchorwa District) of Uganda.. Acta Tropica.

[15] De Lalla F, Zanoni P, Lunetta Q, Moltrasio G (1988). Endemic non-filarial elephantiasis in Iringa District, Tanzania: a study of 30 patients.. Trans R Soc Trop Med Hyg.

[16] Crivelli P (1986). Non-filarial elephantiasis in Nyambene range: a geochemical disease.. East Afr Med J.

[17] Ruiz L, Campo E, Corachan M (1994). Elephantiasis in Sao Tome and Principe.. Acta Tropica.

[18] Corachan M, Tura JM, Campo E, Soley M, Traveria A (1988). Podoconiosis in Equatorial Guinea. Report of two cases from different geological environments.. Trop Geogr Med.

[19] Alemu G, Tekola F, Daniel T, Ahrens C, Davey G (2011). Burden of Podoconiosis in Poor Rural Communities in Gulliso woreda, West Ethiopia.. PLoS NTD.

[20] Oomen AP (1969). Studies on elephantiasis of the legs in Ethiopia.. Trop Geogr Med.

[21] Price EW (1974). Endemic Elephantiasis of the Lower Legs in Ethiopia an Epidemiological Survey.. Ethiop Med J.

[22] Mengistu G, Humber DP, Ersumo M, Mamo T (1987). High prevalence of elephantiasis in Ocholo, south-west Ethiopia.. Ethiop Med J.

[23] Kloos H, Bedri A, Addus A (1992). Podoconiosis (endemic non-filarial elephantiasis) in two resettlement schemes in western Ethiopia.. Tropical Doctor.

[24] Geshere Oli G, Tekola F, Petros B (2012). Clinical, parasitological, and serologic evidence for high prevalence of podoconiosis (non-filarial elephantiasis) in Midakegn district, central Ethiopia.. Trop Med Int Health.

[25] WHO (2010). UN-Water Global Annual Assessment of Sanitation and Drinking - Water (GLAAS).

[26] Yakob B, Deribe K, Davey G (2010). Health professionals' attitudes and misconceptions regarding podoconiosis: potential impact on integration of care in southern Ethiopia.. Trans R Soc Trop Med Hyg.

[27] Yakob B, Deribe K, Davey G (2008). High levels of misconceptions and stigma in a community highly endemic for podoconiosis in southern Ethiopia.. Trans R Soc Trop Med Hyg.

[28] Tora A, Davey G, Getnet Tadelea G (2011). A qualitative study on stigma and coping strategies of patients with podoconiosis in Wolaita zone, Southern Ethiopia.. International Health.

[29] Tekola F, H.Mariam D, Davey G (2006). Economic costs of endemic non-filarial elephantiasis in Wolaita Zone, Ethiopia.. Trop Med Int Health.

[30] CSA, and ORC Macro (2007). The 2007 Population and Housing Census of Ethiopia Population and Housing Census.

